# *PAX2* is dispensable for *in vitro* nephron formation from human induced pluripotent stem cells

**DOI:** 10.1038/s41598-017-04813-3

**Published:** 2017-07-03

**Authors:** Yusuke Kaku, Atsuhiro Taguchi, Shunsuke Tanigawa, Fahim Haque, Tetsushi Sakuma, Takashi Yamamoto, Ryuichi Nishinakamura

**Affiliations:** 1 Department of Kidney Development, Institute of Molecular Embryology and Genetics, Kumamoto University, Kumamoto, Japan; 2 Department of Mathematical and Life Sciences, Graduate School of Science, Hiroshima University, Hiroshima, Japan

## Abstract

The kidney is formed by reciprocal interactions between the nephron progenitor and the ureteric bud, the former of which gives rise to the epithelia of nephrons consisting of glomeruli and renal tubules. The transcription factor *PAX2* is essential for this mesenchymal-to-epithelial transition of nephron progenitors, as well as ureteric bud lineage development, in mice. *PAX2* mutations in humans cause renal coloboma syndrome. We previously reported the induction of nephron progenitors and three-dimensional nephron structures from human induced pluripotent stem (iPS) cells. Here we generate iPS cells lacking *PAX2*, and address the role of *PAX2* in our *in vitro* induction protocol. While *PAX2*-null human nephron progenitors were properly formed, they unexpectedly became epithelialised to form glomeruli and renal tubules. However, the mutant glomerular parietal epithelial cells failed to transit to the squamous morphology, retaining the shape and markers of columnar epithelia. Therefore, *PAX2* is dispensable for mesenchymal-to-epithelial transition of nephron progenitors, but is required for morphological development of glomerular parietal epithelial cells, during nephron formation from human iPS cells *in vitro*.

## Introduction

The mammalian kidney, the metanephros, is formed by reciprocally inductive interactions between two precursor tissues, the metanephric mesenchyme and the ureteric bud^1^. The metanephric mesenchyme gives rise to glomeruli and renal tubules, while the ureteric bud branches to create collecting ducts and ureters, thus forming the nephron, a functional unit of the kidney. The metanephric mesenchyme contains nephron progenitors that undergo mesenchymal-to-epithelial transition upon induction by the ureteric bud. The progenitors sequentially transit to renal vesicles, C-shaped bodies, and S-shaped bodies, eventually forming glomeruli and renal tubules. The proximal region of S-shaped bodies becomes the glomeruli and proximal renal tubules, while the distal region becomes the distal renal tubules. The glomerular epithelia are further segregated into two lineages: visceral epithelial cells (podocytes) and parietal epithelial cells (Bowman’s capsule epithelial cells). Podocytes exhibit a unique morphology, having multiple cellular processes bridged by slit diaphragms, which are filtration apparatuses consisting of transmembrane proteins including NEPHRIN^2^. In contrast, glomerular parietal cells become flattened and adopt a squamous shape. Urine, which is filtrated through the slit diaphragms of the podocytes, flows into the space surrounded by the glomerular parietal epithelial cells (Bowman’s capsule), and then into the adjacent proximal renal tubules, distal renal tubules, collecting ducts, and ureters.

There is an *ex vivo* culture system available to assess the competence of the metanephric mesenchyme. In the system, isolated metanephric mesenchyme is co-cultured with embryonic spinal cord, and the nephron progenitors in the mesenchyme undergo mesenchymal-to-epithelial transition to form nephron structures, including glomeruli and renal tubules. In this setting, the spinal cord functions as a substitute for the ureteric bud, as both can secrete Wnt ligands and induce differentiation of nephron progenitors^3^. Many mutant mice have been analysed using this spinal cord recombination system^4–6^.

Paired box (PAX) genes are homologues of the *Drosophila* pair rule gene *paired* and encode nuclear proteins characterised by DNA-binding paired box domains^7, 8^. The *PAX* family has nine members in mammals, and is categorised into four paralogue groups. *PAX2* constitutes one of the groups with *PAX5* and *PAX8*, and plays important roles in organ development. In the developing mouse kidney, Pax2 is expressed in the nephron progenitors, renal vesicles, S-shaped bodies, and distal renal tubules. *Pax2* deletion in mice leads to kidney agenesis, as well as defects in the genital tract, optic nerve, retina, inner ear, and midbrain^9, 10^. When *Pax2*-deficient metanephric mesenchyme is isolated and co-cultured with spinal cord, no tubulogenesis or nephron formation occurs^11^, indicating an indispensable role of mouse *Pax2* in mesenchymal-to-epithelial transition of nephron progenitors. *Pax2* is also expressed in the ureteric bud, another precursor population of the kidney, and is important for its development. During the initial phase of kidney development, the nephric duct (Wolffian duct) is formed and elongates caudally, followed by sprouting out of the ureteric bud. *Pax2* deficiency causes impaired epithelial integrity of the nephric duct, thereby affecting proper ureteric budding^12^, which is likely to contribute to the kidney agenesis observed in the absence of *Pax2*.

In humans, *PAX2* frameshift mutations cause renal coloboma syndrome, an autosomal dominant disorder manifesting as kidney hypoplasia and various optic nerve defects, accompanied by auditory and central nervous abnormalities in some cases^13, 14^. Heterozygous *Pax2* ^*1Neu*^ mice, which harbour a frameshift mutation found in some human patients, exhibit similar phenotypes^15^, and genetically-engineered *Pax2* heterozygous mice show kidney hypoplasia^9^. These data indicate that the renal coloboma phenotype is caused by *PAX2* haploinsufficiency. This is likely due, in large part, to a role of *PAX2* in the ureteric bud, because the kidney size in heterozygous *Pax2* ^*1Neu*^ mice is restored by apoptosis suppression in the ureteric bud^16^. However, the precise role and expression patterns of *PAX2* in human kidney development are not fully understood, despite the accumulated findings in mice.

We and others previously reported the induction of nephron progenitors from human induced pluripotent stem (iPS) cells^17–20^. In our protocol, the induced progenitor aggregates form three-dimensional glomeruli and renal tubules *ex vivo* upon spinal cord recombination^17, 18^, thus mimicking the situation in murine progenitors. Thus, our protocol should serve as a useful tool to analyze the role of *PAX2* in differentiation of nephron progenitors in humans. For this purpose, we disrupted the *PAX2* gene in human iPS cells by homologous recombination using transcription activator-like effector nucleases (TALENs)^21^, and unexpectedly found a dispensable role of *PAX2* under our nephron differentiation conditions.

## Results

### Generation of *PAX2*-deficient human iPS cells

**Figure 1:**
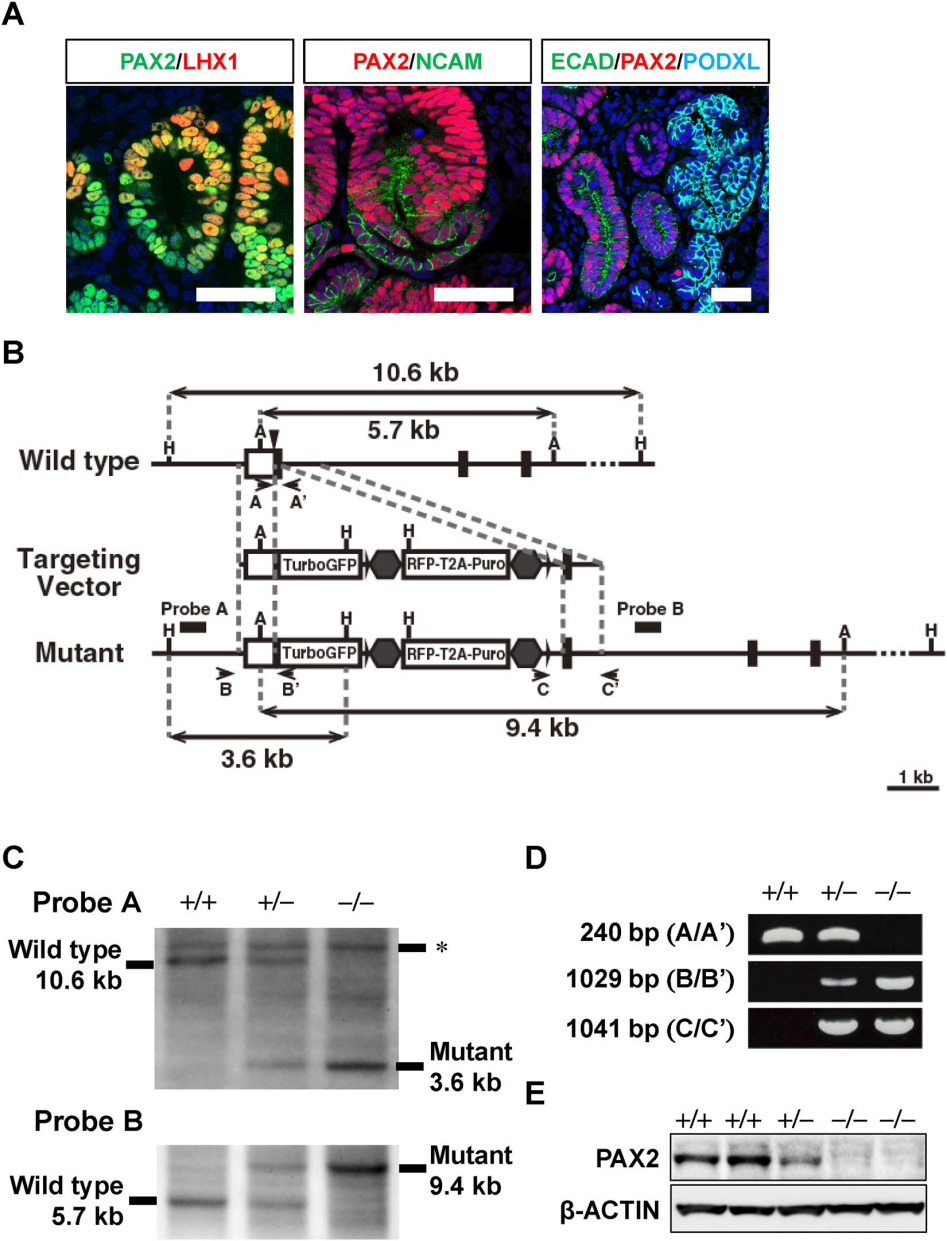
Generation of *PAX2*-deficient human iPS cells. (**A**) PAX2 staining during nephron formation from the parental iPS clone. PAX2 is expressed in the renal vesicles (left panel: green), NCAM^+^ S-shaped bodies (middle panel: green), and E-cadherin (ECAD)^+^ distal renal tubules (right panel: green). LHX1 (left panel: red) is expressed in a portion of renal vesicles, and podocalyxin (PODXL; right panel: blue) is expressed in glomeruli. Scale bars: 50 µm. (**B**) Strategy for targeting the human *PAX2* gene. The *TurboGFP* and *RFP* cassettes are inserted into the *PAX2* locus. The *RFP* and *Puro* cassettes are sandwiched by loxP (arrowheads) and insulator sequences (hexagons). A, *ApaL*I; H, *Hind*III. (**C**) Southern blot analysis of wild-type, heterozygous (+/−), and homozygous (−/−) clones. Genomic DNAs were digested with *Hind* III (upper panel) or *ApaL*I (lower panel) and hybridised with the indicated probes. The positions of the probes are indicated in (**B**). *Non-specific bands. (**D**) PCR screening of homologous recombinants. The positions of the primers are indicated in (**B**). (**E**) Western blot analysis of PAX2 in nephron progenitors derived from iPS clones.

First, we confirmed PAX2 expression during *in vitro* nephrogenesis from human iPS cells. PAX2 was detected in the renal vesicles, S-shaped bodies, and distal renal tubules (Fig. 1A), similar to the expression pattern in mouse embryonic kidneys. To examine the role of PAX2 in human kidney development, we inserted a gene encoding green fluorescent protein (TurboGFP) into the *PAX2* locus of human iPS cells by homologous recombination (Fig. 1B). For this, we constructed a pair of plasmids that expressed TALENs targeting sequences in close proximity to the *PAX2* start codon, and introduced these plasmids, along with a targeting vector expressing the homology arms, into human iPS cells. We successfully obtained heterozygous and homozygous *GFP* knock-in clones (−/−), determined by PCR and Southern blotting (Fig. 1C,D). Subsequently, we induced the correctly targeted clones toward nephron progenitors, and confirmed the reduction and absence of PAX2 protein in the heterozygous and homozygous clones, respectively (Fig. 1E).

**Figure 2:**
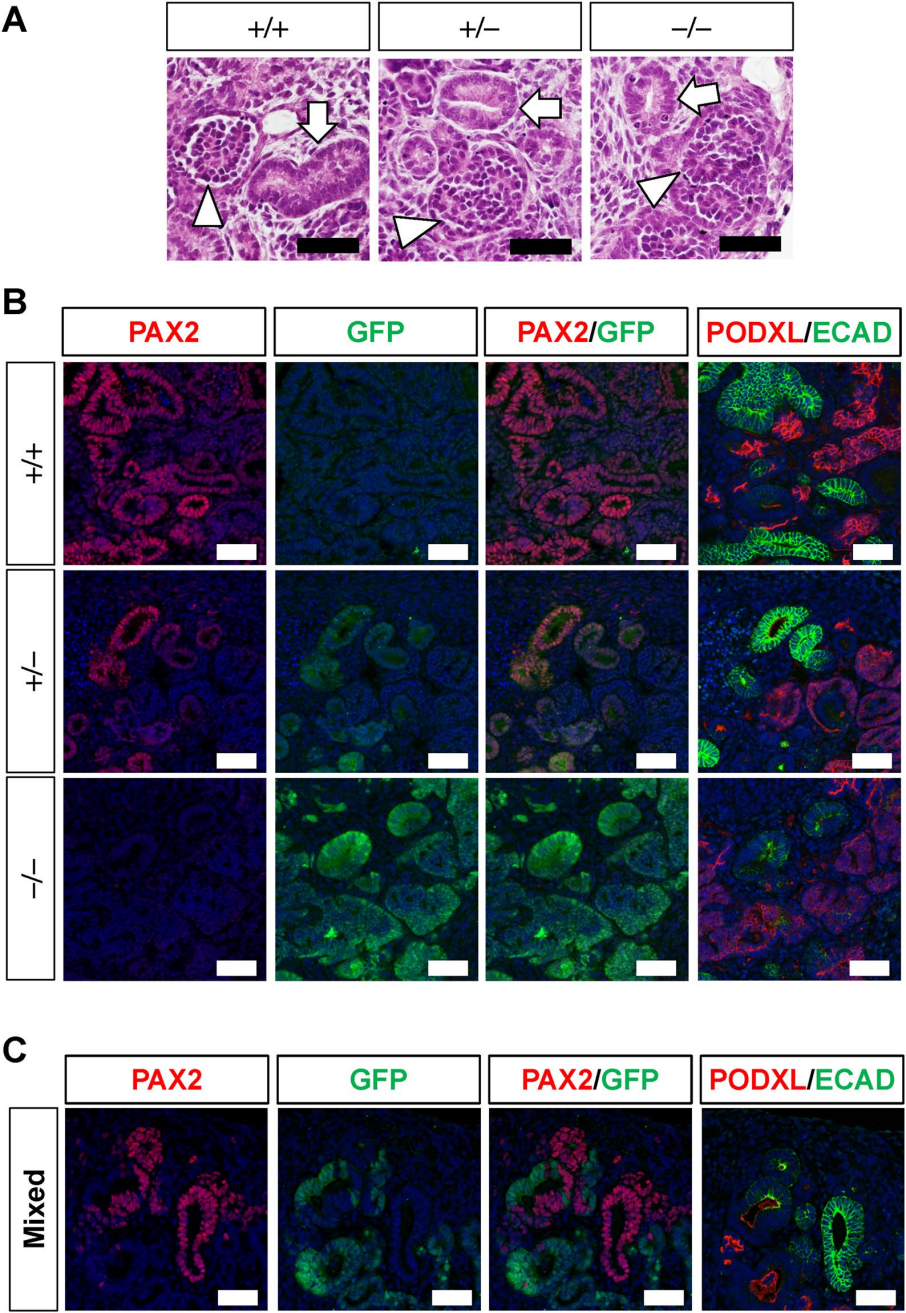
Successful deletion of *PAX2* in renal tubules derived from iPS cells. (**A**) Hematoxylin–eosin (HE) staining of kidney tissues induced from wild-type (+/+), heterozygous (+/−), and homozygous (−/−) clones. Arrowheads: glomeruli; arrows: renal tubules. (**B**) PAX2 (red) and GFP (green) staining of kidney tissues induced from the three genotypes. Rightmost columns: neighbouring sections were stained with markers for renal tubules (E-cadherin: ECAD) and glomerular podocytes (podocalyxin: PODXL). (**C**) Nephron formation from a mixed colony of iPS cells. Nephron epithelia are composed of PAX2^+^ wild-type and GFP^+^ mutant cells. Scale bars: 50 µm.

Next, we co-cultured the induced nephron progenitors with murine spinal cord, as a potent inducer of nephrogenesis, based on our previous reports^17, 18^. At day 9 of co-culture, round-shaped glomeruli and renal tubules with a clear lumen were detected in all three genotypes histologically (Fig. 2A). While PAX2, but not GFP, was detected in wild-type renal tubules, heterozygous clones exhibited weak but faithful GFP expression relative to the expression of endogenous PAX2 (Fig. 2B). In the homozygous mutant clones, a clear GFP signal, but no PAX2 signal, was detected in the renal tubules, indicating successful deletion of PAX2 with compensatory expression of GFP. The GFP signal at the periphery of the glomeruli may reflect PAX2 expression in the glomerular parietal epithelial cells. The successful nephron formation in the absence of human *PAX2* was unexpected, because no tubulogenesis occurred when *Pax2*-deficient mouse embryonic metanephric mesenchyme (containing nephron progenitors) was co-cultured with spinal cord^11^.

During the course of the gene targeting, we obtained one cell line that consisted of both wild-type and homozygous mutant cells, probably through picking up two adjacent colonies. The kidney tissues induced from this mixed colony consisted of either PAX2^+^ or GFP^+^ epithelia (Fig. 2C). These findings indicate that cells lacking PAX2 can differentiate into nephron epithelia even in a competitive environment with wild-type cells, again supporting a dispensable role of human PAX2 in our *in vitro* differentiation protocol.

### Nephron progenitors are properly formed from *PAX2*-null iPS cells

**Figure 3:**
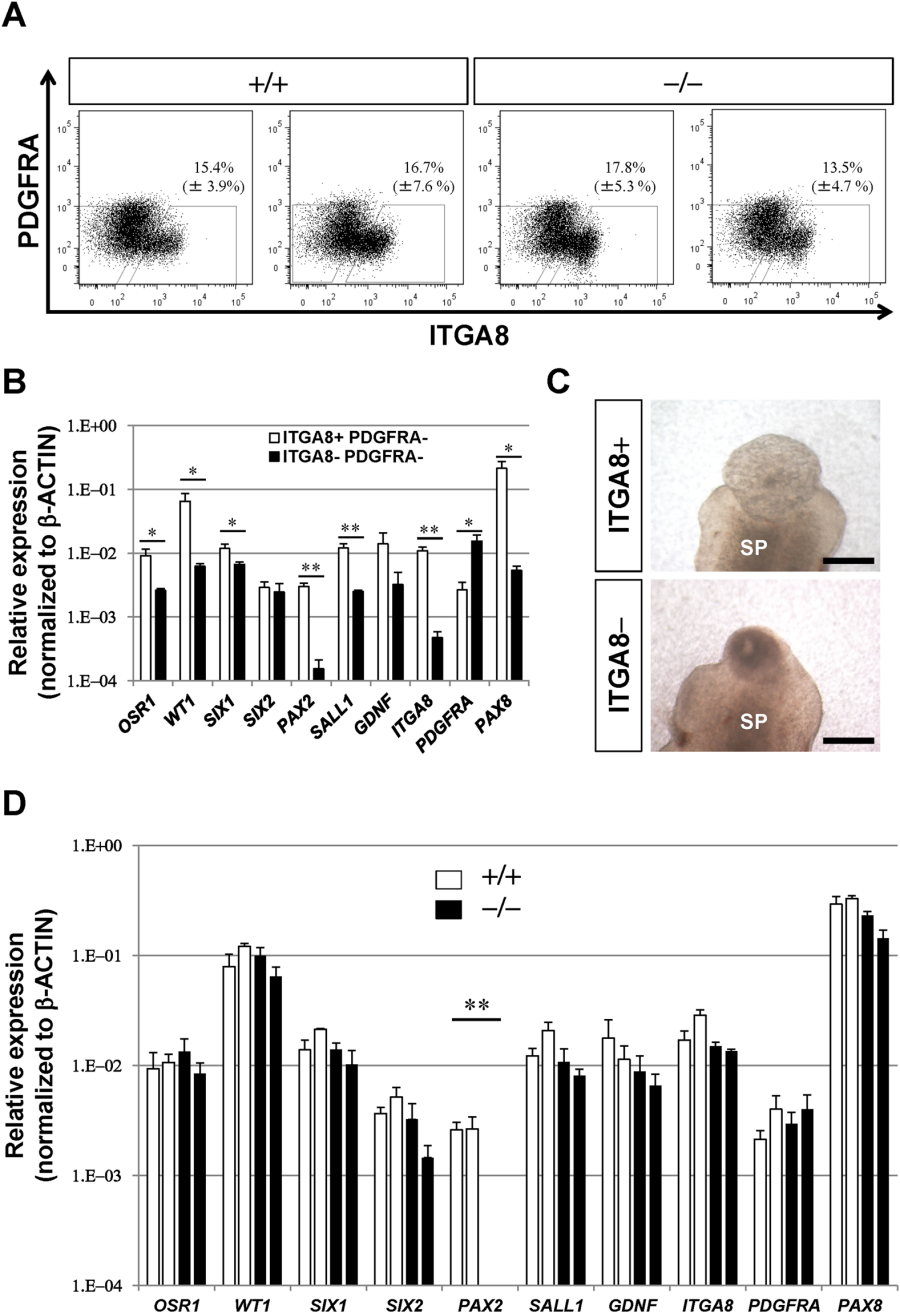
Nephron progenitors are generated from *PAX2*-null iPS cells. (**A**) FACS analysis of tissues induced from wild-type (+/+) and *PAX2*-null (−/−) iPS cells. The percentages of the ITGA8^+^/PDGFRA− nephron progenitor fraction from three independent induction experiments are determined (mean ± standard error). (**B**) Nephron progenitor markers are more abundantly expressed in the ITGA8^+^/PDGFRA^−^ fraction (open bars) than in the ITGA8^−^/PDGFRA^−^ fraction (closed bars) induced from the wild-type clone. Data represent mean ± standard error of three independent induction experiments. *p < 0.05, **p < 0.01. (**C**) Tubulogenesis is observed when the ITGA8^+^/PDGFRA^−^ fraction is co-cultured with spinal cord for 9 days. SP: spinal cord. Scale bars: 200 µm. (**D**) Expression levels of nephron progenitor markers are unaltered, except for *PAX2*. qPCR analyses of sorted ITGA8^+^/PDGFRA^−^ fractions from two wild-type clones (open bars) and two *PAX2*-null clones (closed bars) are shown (mean ± standard error). **p < 0.01.

Nephron formation *in vitro* can be divided into two steps: nephron progenitor induction from iPS cells and subsequent nephron formation from the progenitors. To analyse these two steps precisely, we developed a method to isolate the nephron progenitors, thereby enabling examination of both the induction efficiency of nephron progenitors and the nephron formation from the purified progenitors from each clone. We previously reported that nephron progenitors in mouse embryos can be sorted by the presence of integrin α8 (ITGA8) and absence of PDGF receptor α (PDGFRA)^17^. Meanwhile, O’Brien *et al*.^22^ showed that human embryonic nephron progenitors are also ITGA8-positive. Therefore, we analysed the nephron progenitors induced from one of our wild-type clones (+/+) by FACS analysis, and found that 15.4% of the cells constituted the ITGA8^+^/PDGFRA^−^ population (Fig. 3A). This population expressed higher levels of multiple nephron progenitor markers than the ITGA8^−^/PDGFRA^−^ population, including *OSR1*, *WT1*, *SIX1*, *PAX2*, and *SALL1* (Fig. 3B). *GDNF* was also enriched, although the difference was not statistically significant. Importantly, when co-cultured with murine spinal cord, the ITGA8^+^/PDGFRA^−^ population exhibited robust tubulogenesis, in contrast to the ITGA8^−^/PDGFRA^−^ population (Fig. 3C). Thus, nephron progenitors were enriched in the ITGA8^+^/PDGFRA^−^ fraction. However, the observation that *SIX2* was not enriched in the ITGA8^+^/PDGFRA^−^ fraction indicated that this population may not consist solely of nephron progenitors. We then examined another wild-type clone (+/+) and two *PAX2*-null clones (−/−), and observed variable percentages of the ITGA8^+^/PDGFRA^−^ fraction depending on the experiments (Fig. 3A and Supplementary Table S1), albeit with no significant differences (+/+ clone 1 vs. −/− clone 1: p = 0.34; +/+ clone 1 vs. −/− clone 2: p = 0.40; +/+ clone 2 vs. −/− clone 1: p = 0.46; +/+ clone 2 vs. −/− clone 2: p = 0.35). These experimental variations, which were not apparent when using the parental iPS cells^17^, may partly result from the extensive passages required to establish the genetically manipulated clones. Nevertheless, the sorted fractions expressed similar levels of nephron progenitor markers, with the exception of *PAX2* (Fig. 3D). Although *GDNF* was reported to be a target of *PAX2* ^11^, the expression levels of *GDNF* were not significantly reduced in the *PAX2*-null nephron progenitors (+/+ clone 1 vs. −/− clone 1: p = 0.19; +/+ clone 1 vs. −/− clone 2: p = 0.13; +/+ clone 2 vs. −/− clone 1: p = 0.10; +/+ clone 2 vs. −/− clone 2: p = 0.10). These data indicate that nephron progenitors can be properly induced from human iPS cells in the absence of *PAX2*. These observations are compatible with the situation in mice, given that histologically distinguishable metanephric mesenchyme is formed in *Pax2*-deficient mice^9^.

### Sorted *PAX2*-null human nephron progenitors undergo mesenchymal-to-epithelial transition to form glomeruli and renal tubules

**Figure 4:**
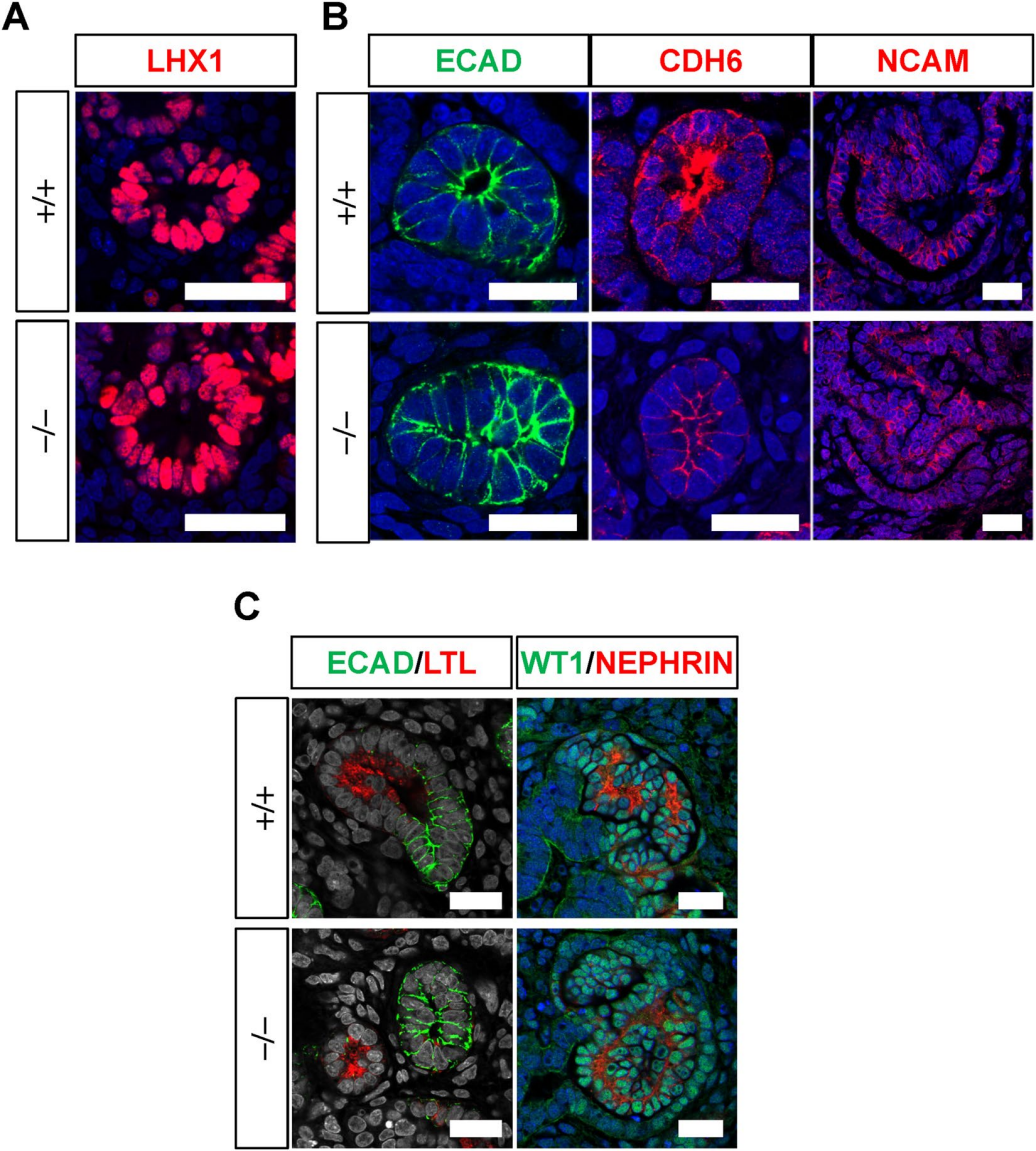
Sorted *PAX2*-null human nephron progenitors undergo mesenchymal-to-epithelial transition. (**A**) LHX1^+^ renal vesicles are induced from both the wild-type and PAX2-null nephron progenitors at day 3 of co-culture with spinal cord. (**B**) E-CADHERIN (ECAD)^+^ distal and CADHERIN6 (CDH6)^+^ proximal renal tubules, as well as NCAM^+^ S-shaped bodies, are formed from the wild-type and *PAX2*-null nephron progenitors at day 6. (**C**) Renal tubules and glomeruli are formed at day 9. Left panels: ECAD^+^ distal and LTL^+^ proximal renal tubules; right panels: WT1/NEPHRIN^+^ glomeruli. Scale bars: 25 µm.

The isolation of nephron progenitor fractions enabled precise comparisons of their competence. The same numbers of ITGA8^+^/PDGFRA^−^ nephron progenitors from wild-type (+/+) and *PAX2*-null (−/−) clones were co-cultured with spinal cord, and their development into nephron structures was examined histologically. At 3 days of co-culture, renal vesicles, marked with Lim Homeobox 1 (LHX1), were formed both in the wild-type and mutant clones (Fig. 4A). At 6 days of co-culture, CADHERIN6^+^ proximal renal tubules and E-CADHERIN^+^ distal tubules were observed from both genotypes (Fig. 4B). These data indicate that mesenchymal-to-epithelial transition, as well as establishment of proximo-distal polarity, was not impaired in the absence of human *PAX2*. In addition, NCAM^+^ S-shaped bodies, of which the proximal regions are precursors of glomeruli, were observed in both the control and mutant clones. At 9 days of co-culture, proximal (Lotus tetragonolobus lectin: LTL^+^) and distal (E-CADHERIN^+^) renal tubules were formed (Fig. 4C). In addition, the glomeruli showed WT1^+^ podocytes with basally-distributed NEPHRIN expression (Fig. 4C). Therefore, nephron formation was comparable between the wild-type and *PAX2*-mutant clones, even when started from the same numbers of nephron progenitors, clearly indicating the dispensable role of *PAX2* in mesenchymal-to-epithelial transition of nephron progenitors in the differentiation process from human iPS cells.

### Human *PAX2* is required for morphological development of glomerular parietal epithelial cells

**Figure 5:**
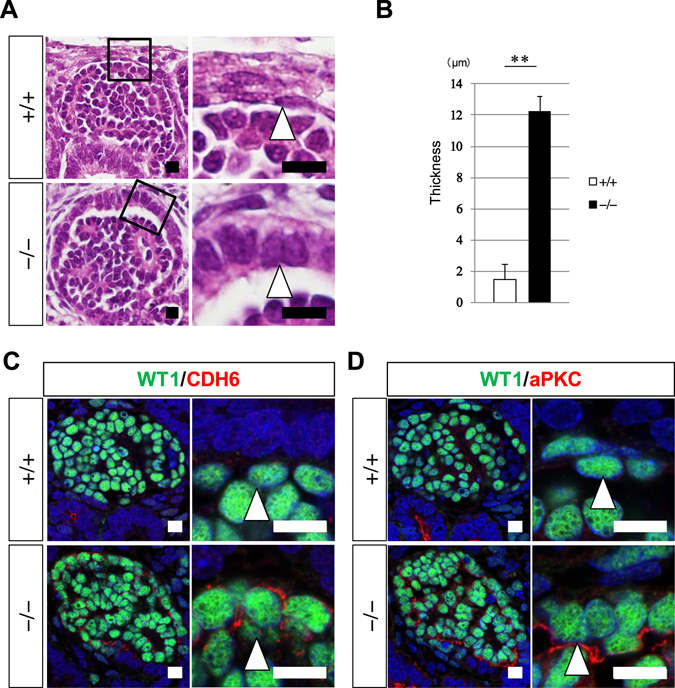
Human *PAX2* is required for morphogenesis of glomerular parietal epithelial cells. (**A**) Hematoxylin–eosin staining of glomerular parietal epithelial cells from the wild-type and *PAX2*-null iPS cells. Note the flat (+/+) and columnar (−/−) shapes. Right panels: magnified images of the squares. (**B**) Thickness of the glomerular parietal epithelial cells (mean ± standard error). **p < 0.01. (**C**) CADHERIN6 (CDH6) is only expressed in the mutant glomerular parietal epithelia. (**D**) aPKC is only expressed in the mutant glomerular parietal epithelia. Arrows: glomerular parietal epithelial cells. Scale bars: 10 µm.

Despite the apparently normal glomerulogenesis in the absence of *PAX2*, we noticed that the mutant glomerular parietal epithelial cells (epithelia of Bowman’s capsule) were not flattened and remained in the columnar shape (Fig. 5A). The thickness of the epithelial layers was significantly larger in the mutant clones compared with the wild-type clones (Fig. 5B). In addition, CADHERIN6, which showed restricted expression to columnar proximal renal tubules in the wild-type clone, was detected in the basolateral domains of the mutant glomerular parietal epithelial cells (Fig. 5C). Furthermore, atypical protein kinase C (aPKC), which is normally expressed in the apical domains of columnar epithelia, was detected in the mutant glomerular parietal cells (Fig. 5D). Therefore, *PAX2*-deficient glomerular parietal epithelial cells failed to undergo columnar-to-squamous transition, and retained columnar marker expression, suggesting a requirement for human *PAX2* in the morphological development of this cell lineage.

### PAX8 expression domains are expanded in *PAX2*-null renal vesicles

**Figure 6:**
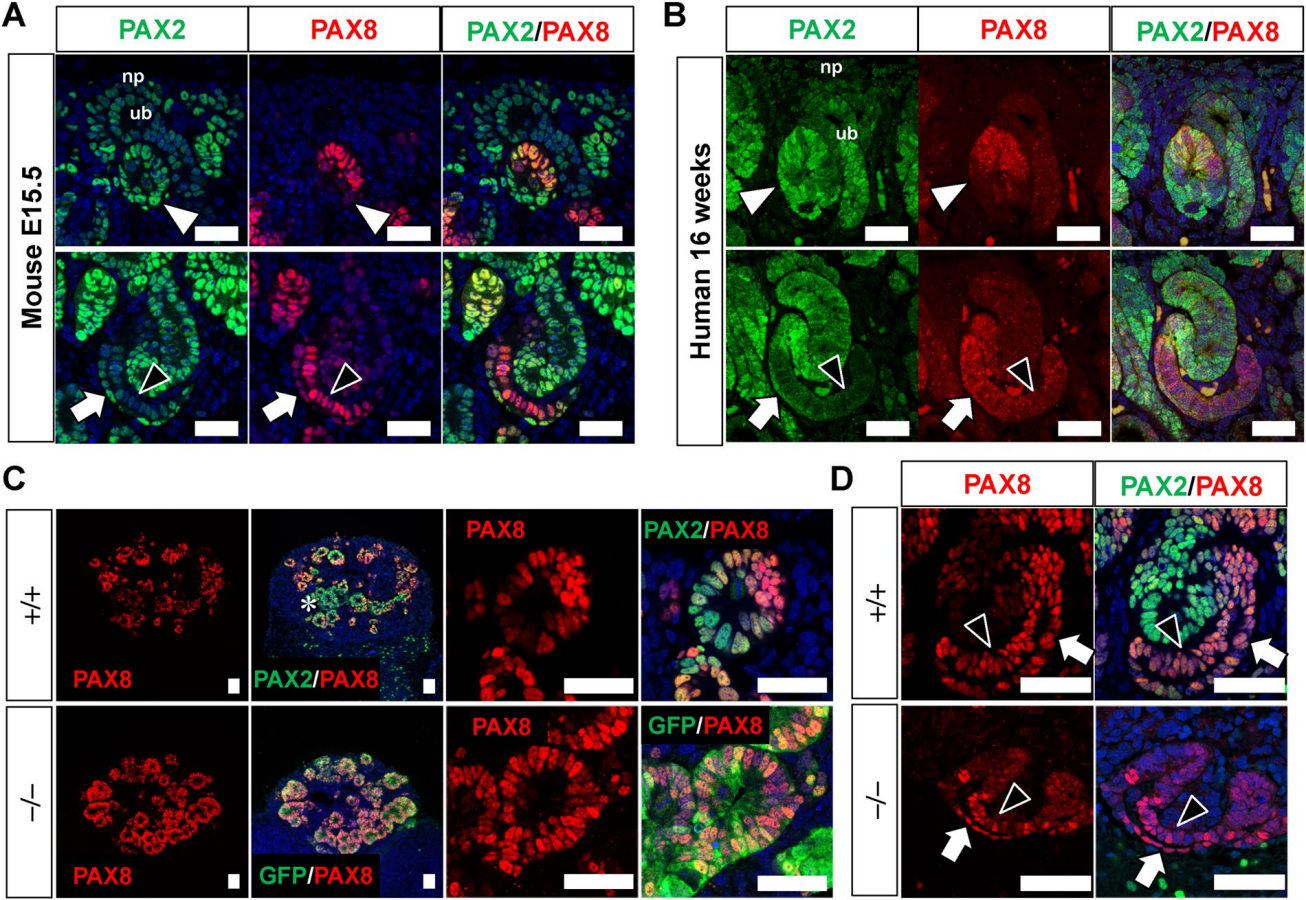
PAX8 expression domains are expanded in *PAX2*-null human renal vesicles. (**A**) Expression of PAX2 and PAX8 in the mouse kidney at E15.5. Upper panels: renal vesicles; lower panels: S-shaped bodies. (**B**) Expression of PAX2 and PAX8 in the human kidney at 16 weeks of gestation. Upper panels: renal vesicles; lower panels: S-shaped bodies. (**C**) Expression of PAX8 in renal vesicles induced from the wild-type and *PAX2*-null iPS cells. In the latter case, GFP staining was used to identify the cells that should have expressed PAX2. Note that PAX8 is expressed in most of the GFP^+^ epithelia, while PAX2^+^/PAX8^−^ vesicles were detected in the wild-type epithelia (asterisk). (**D**) Expression of PAX2 and PAX8 in S-shaped bodies induced from the wild-type and *PAX2*-null iPS cells. White arrowheads: renal vesicles; arrows: precursors of glomerular parietal epithelia; black arrowheads: precursors of podocytes. np: nephron progenitors; ub: ureteric bud. Scale bars: 50 µm.

The unimpaired mesenchymal-to-epithelial transition under *PAX2* deficiency may be explained through compensation by other redundant genes. In mice, *Pax8*, which belongs to the same *Pax* paralogue group as *Pax2*, functions redundantly with *Pax2* in kidney development, whereas *Pax5* is not expressed in the kidney. While *Pax8* deletion alone exhibits no apparent phenotypes, *Pax2*/*Pax8* deficiency causes defects in nephric duct formation, which occurs much earlier than metanephros formation^23, 24^. During metanephros formation, *Pax8* also functions redundantly with *Pax2*, because *Pax2*/*Pax8* double-heterozygous mice exhibit more severe kidney hypoplasia than *Pax2* heterozygous mice^23, 25^. As determined by *in situ* hybridisation analyses, mouse *Pax8* is expressed in renal vesicles and S-shaped bodies, but not in the metanephric mesenchyme (nephron progenitors) where *Pax2* exists^25^. We found that one of the commercially available anti-PAX8 antibodies showed consistent results with the reported patterns obtained by *in situ* hybridisation^25^. By using this anti-PAX8 antibody, we confirmed that PAX2, but not PAX8, was expressed in the murine metanephric mesenchyme and ureteric buds, while both PAX2 and PAX8 were expressed in the renal vesicles (Fig. 6A and Supplementary Fig. S1). However, PAX8 expression was confined to the distal part of the renal vesicles, while PAX2 was expressed in all regions. As development proceeded, PAX2 was expressed in the middle and distal parts of the S-shaped bodies (Fig. 6A and Supplementary Fig. S1). In addition, although weak, PAX2 was more abundantly expressed in the precursors of glomerular parietal epithelial cells than in those of podocytes. In contrast, PAX8 was expressed in both lineages, and weakly in the middle part of the S-shaped bodies. These expression patterns were almost conserved in human embryonic kidneys (Fig. 6B). PAX2 was detected in the metanephric mesenchyme (nephron progenitors) and the entire regions of the renal vesicles, as well as in the ureteric bud tips. Weak PAX2 expression was also observed in the precursors of glomerular parietal cells. PAX8 was expressed in the distal region of renal vesicles, middle part of S-shaped bodies, and precursors of glomerular podocytes and parietal cells.

We further examined the expression pattern of PAX8 during nephrogenesis from the wild-type and *PAX2*-null human iPS cells. Our qPCR analysis detected *PAX8* expression in the ITGA8^+^/PDGFRA^−^ progenitor fraction (Fig. 3B). However, its expression level was not high, given the absence of PAX8 immunostaining in the nephron progenitor region (Fig. 6B). Moreover, *PAX8* expression in *PAX2*-null progenitors was unaltered (Fig. 3D). At the renal vesicle stage, PAX2 was expressed in most of the wild-type epithelia, while PAX8 was expressed in a subset of PAX2^+^ epithelia (Fig. 6C). There were PAX2-single-positive renal vesicles, and even in PAX2^+^/PAX8^+^ vesicles, PAX8^high^ cells were clustered at one end, consistent with the findings *in vivo*. In the *PAX2* mutant clones, we stained GFP to detect the cells with *PAX2* promoter activity, and found that it marked most of the epithelia. Interestingly, PAX8 was detected in most of the PAX2^+^ epithelia (Fig. 6C), suggesting compensatory expression of PAX8 in the absence of PAX2. However, at the S-shaped body stage, PAX2, and more abundantly PAX8, were expressed in the precursors of glomerular podocytes and parietal cells in the wild-type epithelia (Fig. 6D), and there was no compensatory PAX8 expression in the mutant epithelia. Thus, the compensatory expression of PAX8 upon *PAX2* deletion in the renal vesicles may explain the successful mesenchymal-to-epithelial transition to form nephrons, whereas failure of such compensation in the glomerular parietal cells could lead to the morphogenetic abnormalities of this cell lineage.

## Discussion

We have examined the role of *PAX2* in human nephron formation, by utilizing an induction protocol for nephron progenitors from iPS cells. By generating homozygous *PAX2*-null iPS cells, we have demonstrated that *PAX2* is dispensable for the formation of nascent nephrons. These findings are significantly different from those obtained in *Pax2*-deficient mice. In mice, *Pax2* is expressed in both lineages derived from nephron progenitors and the ureteric bud. *Pax2*/*Pax8* double-heterozygous mice, as well as *Pax2*/*Wt1* double-heterozygous mice, show more severe renal hypoplasia than *Pax2* heterozygous mice^23, 25, 26^. Because *Pax8* and *Wt1* are exclusively expressed in the metanephric mesenchyme (including nephron progenitors), *Pax2* is likely to have a role in this cell lineage, in addition to the ureteric bud. Indeed, mouse *Pax2*-null metanephric mesenchyme co-cultured with spinal cord fails to undergo mesenchymal-to-epithelial transition, thereby leading to a lack of nephron formation^9^. In contrast, human iPS cell-derived *PAX2*-null nephron progenitors in the same setting successfully epithelialise to form nephrons. This may be due to the compensatory expression of other genes at the mesenchymal-to-epithelial transition stage, including PAX8, because the PAX8 expression domain is expanded in the *PAX2*-null renal vesicles. Meanwhile, *PAX8* is not up-regulated in the *PAX2*-null nephron progenitors, the stage before epithelial transition occurs. Thus, deletion of both *PAX2* and *PAX8* is required to prove the compensatory role of *PAX8*. Nonetheless, it is clear that mesenchymal-to-epithelial transition is less dependent on PAX2 in nephron formation from human iPS cells.

PAX2 is also expressed in precursors of the glomerular parietal epithelia in both humans and mice^27^. However, owing to the severe defects in nephron formation in *Pax2*-deficient mice, the importance of this gene in parietal cells has not been addressed. Based on the unaffected nephron formation from *PAX*2-deficient human nephron progenitors, we have shown that *PAX2* is indeed required for the proper morphology of glomerular parietal cells. Our data revealed sustained expression of characteristic markers of columnar epithelia in *PAX2*-null parietal cells. In particular, the existence of CADHERIN6 suggests that the mutant cells retained the character of adjacent proximal renal tubules. PAX2 may inhibit CADHERIN6 expression directly or indirectly, thereby regulating the morphological changes of the glomerular parietal epithelia.

The discrepant data between human iPS cells and genetically engineered mice should be carefully interpreted, before concluding that there are species-specific differences in the requirement for *PAX2*. First, our protocol for nephron formation *in vitro* may not completely recapitulate nephron formation *in vivo*. As previously demonstrated, nephron-forming competence is retained in progenitors with a broader spectrum of gene expression patterns than expected^28, 29^. Thus, it is possible that nephron formation based on the present protocol is less dependent on *PAX2*. Application of the protocol to *Pax2*-null mouse embryonic stem cells would be helpful to address the species-specific differences in the role of *PAX2*, at least in the *in vitro* setting. However, careful extrapolation is needed for its role in humans *in vivo*. Second, the spinal cord is a potent inducer of nephrogenesis from nephron progenitors, but does not extensively support their propagation, thereby leading to rapid depletion of the progenitors. Thus, the present study may not address the role of *PAX2* in the propagation and maintenance of nephron progenitors. Recently, we and others reported the *in vitro* propagation of nephron progenitors induced from human iPS cells^29–31^. If a complete propagation method can be established, it would be worthwhile applying this method to *PAX2*-null progenitors.

*PAX2* heterozygous iPS cells showed unaltered nephron formation, while accumulating evidence indicates that the renal coloboma phenotype is caused by *PAX2* haploinsufficiency^9, 13, 15^. However, the kidney hypoplasia in heterozygous *Pax2* ^*1Neu*^ mice could largely result from defects in the ureteric bud, because suppression of apoptosis in the ureteric bud restores this phenotype^16^. This notion may be consistent with the unaltered nephrogenesis from heterozygous human iPS cells, because our protocol selectively induces nephron progenitors, but not the ureteric bud. The present study does not address the role of human *PAX2* in the ureteric bud, another important progenitor that constitutes the kidney. The *Pax2*-deficient murine Wolffian duct, as the precursor of the ureteric bud, exhibits impaired epithelial integrity, comprising loss of polarity and reduced intercellular adhesion^12^. Although a few groups have reported protocols for induction of ureteric buds from human iPS cells^32, 33^, the branching capacity and nephron-inducing potential were not sufficient. Thus, more robust protocols for the formation of competent Wolffian ducts/ureteric buds are under development in many laboratories, including ours, and these protocols will be useful for examining the role of *PAX2* in this important lineage.

Regarding the technical aspects of the iPS cell-based differentiation, we established a method to sort human nephron progenitors by utilising ITGA8 expression on the cell surface, which enabled us to precisely compare the competence of nephron progenitors from different iPS clones. Clonal and experimental variations in the differentiation efficiencies of any organs are the bottleneck of iPS cell technology. Even when induction protocols are modified for individual clones, induction efficiencies may still vary depending on the experiments. While several factors, such as gene manipulation, passage numbers, and colony conditions, may cause these variations, the gene expression and differentiation competence of the sorted progenitors were consistent. Our data suggest that differentiation potentials can be precisely testable by starting with purified progenitors derived from individual clones. In this way, the subtle differences caused by gene deletion should be detected over clonal and experimental variations.

Taken together, we have established *PAX2*-deficient human iPS cells and revealed the dispensable role of *PAX2* in nephron formation *in vitro*. Further improvement of the induction methods from human iPS cells will accelerate our understanding of kidney development in humans. Furthermore, human *PAX2* mutations manifest many extra-renal symptoms, including optic nerve colobomas, auditory abnormalities, and in some cases, central nerve malformation^13, 14^. Thus, our *PAX2*-deficient iPS cells will be useful tools to address the role of *PAX2* in various tissues in humans.

## Methods

### Generation of *PAX2-TALEN* plasmids and the targeting vector

*PAX2*-TALENs were designed to bind to the following sequences and cleave close to the start codon of *PAX2*: 5′-TCCTCTGCCTCCCCATGG-3′ for the left TALEN and 5′-CAGACCCCTTCTCCGCGA-3′ for the right TALEN (underlined sequence corresponds to the start codon). A Platinum Gate TALEN Kit (Addgene; #1000000043) was used to construct the TALEN expression vectors as described previously^34^. The *PAX2*-TALEN plasmids were transfected into HEK293 cells using Lipofectamine 2000 (Thermo Fisher Scientific) to evaluate their activity. The target region was amplified with the following primers: 5′-CACCGTCCCTCCCTTTTCT-3′ and 5′-CCAAGATGGGACCTGAGCG-3′ (primers A and A′, respectively). When the resulting fragment was denatured and annealed, a clear band shift was observed, indicating the formation of a mismatched duplex resulting from deletions or insertions^35^.

For the targeting vector, 5′ and 3′ homology arms (0.71 kb and 0.82 kb, respectively) of *PAX2* were amplified from the genomic DNA of iPS cells with the following primers: 5′-TGAATTCTTAGAGAGACACACACCGGG-3′ and 5′-TGCTAGCGGGGAGGCAGAGGAGCGGGA-3′ for the 5′ homology arm, and 5′-AACCTAGATCGGATCTGCACCGTGAGTACCGGCGC-3′ and 5′-ATTACGCCAAGCTTGCTGGCTCTCTCCCTGACTTC-3′ for the 3′ homology arm. Upon sequence verification, the 5′ homology arm was cloned in the *EcoRI-NheI* site of the modified HR120PA-1 vector (Systems Biosciences), such that the start codon of *PAX2* was replaced with that of *TurboGFP*, followed by red fluorescent protein (*RFP*) and puromycin-resistance gene (*Puro*) cassettes flanked by loxP and insulator sequences. The 3′ homology arm was cloned in the *BamHI-SphI* site using an In-fusion HD Cloning Kit (Takara Bio).

### Generation of *PAX2*-deficient iPS cells

The human iPS cell line (201B7) was maintained on mouse embryonic fibroblasts as described^36^. The cells were pre-treated with Y27632 (10 µM) at 1 h prior to electroporation, and dissociated into single cells with dissociation solution (Reprocell; RCHETP002) followed by Accutase (Millipore). The targeting vector (10 µg), as well as the pair of TALEN plasmids (5 µg each), were electroporated into the dissociated human iPS cells using a SuperElectroporator NEPA21 (Nepagene) under the following conditions: two poring pulses (125 V, 2.5 ms) followed by five transfer pulses (20 V, 50 ms). The cells were then plated onto puromycin-resistant DR4 feeders, and puromycin (0.25 µg/ml) was added at 2 days after electroporation^37^. After 2 days of incubation, the puromycin concentration was increased to 0.5 µg/ml. The primers used for PCR screening of 5′ recombination were 5′-CCCATTTCTCCCTCCCCTG-3′ and 5′-CCACCAGCTCGAACTCCA-3′ (primers B and B′, respectively; product size: 1029 bp), and those for 3′ recombination were 5′-GGCTGTCCCTGATATCAAACA-3′ and 5′-GGCTGCGTGATCCTCAATG-3′ (primers C and C′, respectively; product size: 1041 bp). The PCR amplifications were performed as described^18^. The digoxygenin-labelled probes for Southern blot analysis were amplified using a PCR Dig Probe Synthesis Kit (Roche) and the following primers: 5′-AGCTCGATTCTGAACCAAGC-3′ and 5′-GGGAGCCCGGGATTAAAACT-3′ for probe A, and 5′-GGAAAGCCTCGGTCCTTTTC-3′ and 5′-CTCTAGCCCCACTTCTCACC-3′ for probe B. We obtained two homozygous *GFP* knock-in clones (−/−) among 91 clones, as determined by PCR and Southern blotting. Although we obtained 35 candidate heterozygous clones, most of the clones had mutations at the TALEN target sites in the non-GFP-containing allele, probably arising from the high activity of TALEN plasmids, resulting in the acquisition of only one heterozygous clone (+/−). Likewise, only two wild-type clones were confirmed to be devoid of mutations at their TALEN target sites (+/+). All of the clones showed nephron formation, and GFP faithfully mimicked PAX2 expression without deletion of the puromycin-resistance cassette, as shown in Fig. 2. All experiments were approved by the Committee on Living Modified Organisms of Kumamoto University (#A27-064), and performed in accordance with the institutional guidelines of Kumamoto University.

### Induction and isolation of nephron progenitors from *PAX2*-deficient iPS cells

The iPS clones were induced toward nephron progenitors using our previously described method^17, 18^, with a minor modification. At day 0–1, we added BMP4 at 0.25 ng/ml, instead of 0.5 ng/ml, to optimise the percentage of the ITGA8^+^/PDGFRA^−^ nephron progenitor fraction. Sorting of nephron progenitors was performed as described^18^. The antibodies used for staining were as follows: phycoerythrin-conjugated anti-PDGFRA (Biolegend; 323506), biotinylated anti-ITGA8 (R&D Systems; BAF4076), and allophycocyanin-conjugated streptavidin (Biolegend; 405207). Data were obtained using a FACS SORPAria (BD Biosciences) and analysed with FlowJo software (TOMY Digital Biology).

### Kidney induction from *PAX2*-deficient nephron progenitors

The sorted nephron progenitors (50,000 cells/well) were re-aggregated in a V-shaped-bottom non-adherent plate (Sumitomo Bakelite; MS-9096V) for 2 days. We used our previously described medium^29^, except that the Notch inhibitor DAPT was omitted. The re-aggregated nephron progenitors were cultured at the air–fluid interface on a Nuclepore membrane (GE Healthcare; 110409) supplied with DMEM containing 10% foetal calf serum, and with mouse embryonic spinal cord taken from E12.5 embryos^17, 18^. The intact unsorted nephron progenitor spheres were used for the experiments shown in Figs 1 and 2. All animal experiments, including embryonic spinal cord isolation, were approved by the Animal Care and Use Committee of Kumamoto University (#A27-018R1), and performed in accordance with the institutional guidelines of Kumamoto University.

### Immunohistochemical analysis

Samples were fixed in 10% formalin, embedded in paraffin, and cut into 6-μm sections. Antigen retrieval in citrate buffer was performed before staining. The following primary antibodies were used: rabbit anti-PAX2 (Biolegend; 901001); mouse anti-turboGFP (Origene; TA150041); rabbit anti-WT1 (C19) (Santa Cruz Biotechnology; sc-192); anti-LHX1 (Developmental Studies Hybridoma Bank; 4F2); mouse anti-E-cadherin (BD Biosciences; 610181); rabbit anti-cadherin6 (a kind gift from Dr. Gregory Dressler, University of Michigan)^38^; mouse anti-NCAM (Ancell; 208-020); rabbit anti-NCAM (Millipore; AB5032); guinea pig anti-nephrin (Progen; GP-N2); goat anti-podocalyxin (R&D Systems; AF1658); anti-rat anti-cytokeratin-8 (Developmental Studies Hybridoma Bank; TROMA-1); mouse anti-SALL1 (Perseus Proteomics; PP-K9814-00); and rabbit anti-phosphorylated aPKC (Abcam; ab62372). Secondary antibodies were conjugated with Alexa 488 or 568 (Thermo Fisher Scientific). For staining with biotinylated LTL (Vector Laboratories; B-1325), a streptavidin-conjugated secondary antibody (Thermo Fisher Scientific) was used. Immunofluorescence was visualised with an LSM780 confocal microscope (Zeiss) or TCS SP8 confocal microscope (Leica). At least three control and three mutant samples at each time point obtained from three independent induction experiments were serially sectioned and showed consistent results.

For staining of PAX8, sections were treated for antigen retrieval and subsequently incubated with an anti-PAX8 antibody (Abcam; ab53490) followed by an anti-mouse secondary antibody conjugated with peroxidase polymers from an ImmPress Reagent Kit (Vector Laboratories; MP-7402). The sections were then incubated with Alexa Fluor 594-tyramide (Thermo Fisher Scientific) for 15 min at room temperature. The stained expression patterns were consistent with the reported results obtained by *in situ* hybridisation^25^. In contrast, staining with another anti-PAX8 antibody (Proteintech; 10336-1) showed almost identical patterns to those of PAX2, indicating the cross-reactivity of this antibody with PAX family proteins. Sections of human embryonic kidney were purchased from USbiomax, and stained for PAX2 and PAX8 using the ImmPress Reagent Kit and Alexa Fluor-tyramide, as described above.

### Quantitative RT-PCR analysis

RNA was isolated using an RNeasy Plus Micro Kit (Qiagen), and then reverse-transcribed with random primers and a Superscript VILO cDNA Synthesis Kit (Life Technologies). Quantitative PCR was carried out using a Dice Real Time System Thermal Cycler (Takara Bio) and Thunderbird SYBR qPCR Mix (Toyobo). The primer sequences are listed in Supplementary Table S2. For the data shown in Fig. 3B, fractions from three independent experiments were analysed. For the data shown in Fig. 3D, three spheres were combined to represent one sample, and three samples were analysed for each clone. The data were analysed by Student’s *t*-test.

### Western blotting analysis

Total cell lysates were harvested using NuPAGE LDS Sample Buffer (Thermo Fisher). Western blotting was performed as described previously^39^. The following primary antibodies were used: anti-PAX2 (Biolegend; 901001); and anti-β-actin (Cell Signaling Technology; 3700).

### Thickness of glomerular parietal epithelial cells

The thicknesses at five points of the parietal layers of one glomerulus were measured using Photoshop CS6 Extended (Adobe), and averaged. One hundred glomeruli (20 glomeruli/section in five sections) from each genotype were analysed by Student’s *t*-test.

## Electronic supplementary material

Supplemental information
