# Supplementary material for: PAX2 is dispensable for in vitro nephron formation from human induced pluripotent stem cells
Source: Sci Rep. 2017 Jul 3;7:4554. doi: 10.1038/s41598-017-04813-3 (PMC5495778; doi:10.1038/s41598-017-04813-3)
Supplement: Supplementary file 1 — Supplemental information [file 41598_2017_4813_MOESM1_ESM.pdf]

Supplemental information

PAX2 is dispensable for *in vitro* nephron formation from human induced pluripotent stem cells

Yusuke Kaku[1], Atsuhiro Taguchi[1], Shunsuke Tanigawa[1], Fahim Haque[1], Tetsushi Sakuma[2], Takashi Yamamoto[2] & Ryuichi Nishinakamura[1]*

[1]Department of Kidney Development, Institute of Molecular Embryology and Genetics, Kumamoto University, Kumamoto, Japan
[2]Department of Mathematical and Life Sciences, Graduate School of Science, Hiroshima University, Hiroshima, Japan

*** E-mail: ryuichi@kumamoto-u.ac.jp**

Supplementary Figure S1. Mouse nascent nephrons.

(A) Immunostaining of murine renal vesicles at E15.5. The neighbouring section to that shown in the upper panels of Figure 6A was stained to confirm the identity of the ureteric bud, which is positive for cytokeratin-8 (CK8). SALL1 is expressed in the nephron progenitors and the distal part of the renal vesicles. (B) Immunostaining of murine S-shaped bodies at E15.5. The neighbouring section to that shown in the lower panels of Figure 6A was stained for NCAM to clearly show the S-shaped bodies. PAX8 is expressed in the precursors of podocytes and parietal cells. White arrowhead: renal vesicles; arrow: precursors of glomerular parietal epithelia; black arrowhead: precursors of podocytes. np: nephron progenitors; ub: ureteric bud. Scale bars: 50 μm.

Supplementary Figure S1

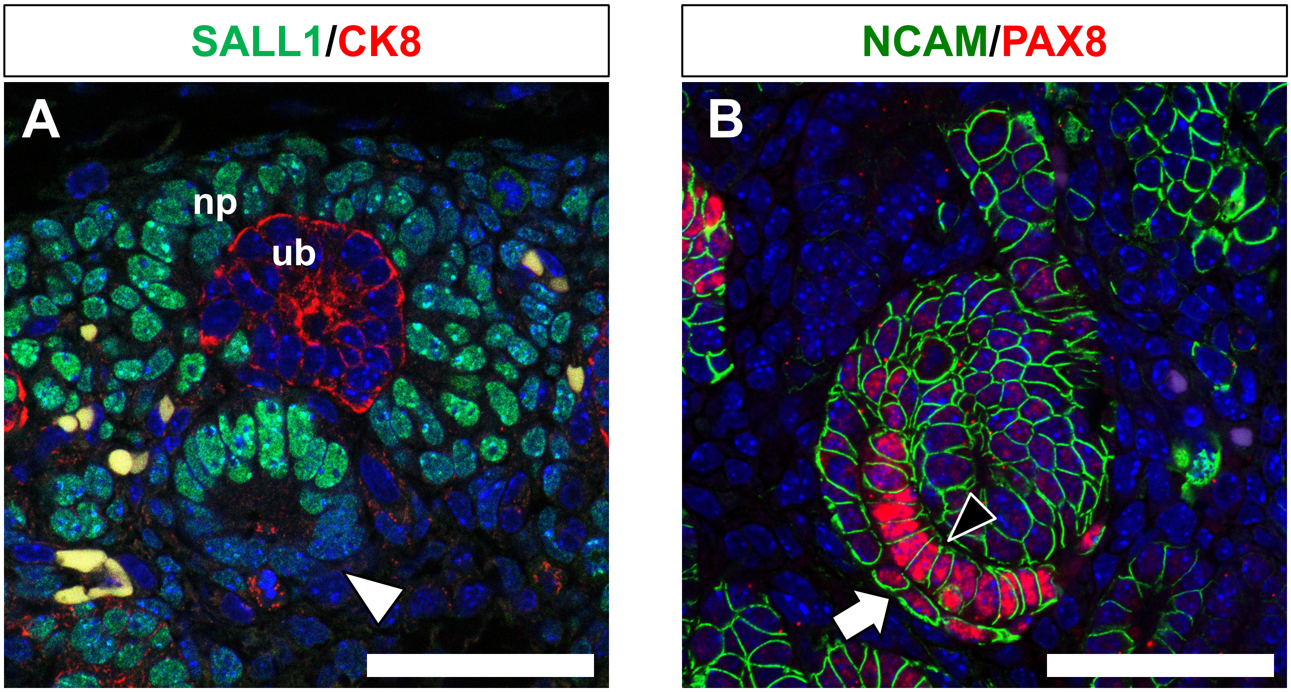

Supplementary Table S1 The percentages of nephron progenitors in the induced spheres from wild-type (+/+) and *PAX2* -null(-/-) clones.

	+/+		-/-	
	Clone 1	Clone 2	Clone 1	Clone 2
Experiment #1	10.5 %	1.8 %	19.8 %	14.9 %
Experiment #2	23.2 %	26.2 %	25.8 %	20.8 %
Experiment #3	12.5 %	22.2 %	7.9 %	4.7 %
Average	15.4 %	16.7 %	17.8 %	13.5 %
Standard Error	3.9 %	7.6 %	5.3 %	4.7 %
	vs +/+ Clone 1		p=0.36	p=0.38
	vs +/+ Clone 2		p=0.45	p=0.36

The percentages of the ITGA8+/PDGFRA− nephron progenitor fraction are shown.

Supplementary Table S2 | Primer sequences used for qPCR.

Primers for quantitative RT-PCR		
Gene	Forward	Reverse
β −ACTIN	TGGCACCCAGCACAATGAA	CTAAGTCATAGTCCGCCTAGAAGCA
OSR1	GACATCTGCCACAAAGCCTTC	CCCACAGGTTCTATTTAGCATTTGA
WT1	AGGGTACGAGAGCGATAACCACAC	CTCAGATGCCGACCGTACAAGA
SIX1	GGTTTAAGAACCGGAGGCAAAG	AGGACCGAGTTCTGGTCTGGA
SIX2	ACAGGTCAGCAACTGGTTCAAGA	ACTTGCCGCTGCCATTCA
PAX2	AACGACAGAACCCGACTATG	ATCCCACTGGGTCATTGGAG
SALL1	TGCAAACAGAATGCAAGCGTTA	AGGCATTGCATCAACACCAGA
GDNF	ACACTGTCTTGGGATCTTGCTGA	TGCTCCACGAATGACTGGATG
ITGA8	GTGCAGTGGGACGACTCGAA	ACACCAGGGATGCAAGAGCATAG
PDGFRA	GTGCGAAGACTGAGCCAGATTG	CGATAAACAGAATGCTTGAGCTGTG
PAX8	CTGAGGGCGTCTGTGACAATG	TGAATGGTTGCTGCACTTTGG
